# The Effects of Maternal and Paternal Body Mass Index on Live Birth Rate after Intracytoplasmic Sperm Injection Cycles

**DOI:** 10.22074/ijfs.2019.5433

**Published:** 2019-01-06

**Authors:** Arezoo Arabipoor, Mahnaz Ashrafi, Mandana Hemat, Zahra Zolfaghari

**Affiliations:** 1 Department of Endocrinology and Female Infertility, Reproductive Biomedicine Research Centre, Royan Institute for Reproductive Biomedicine, ACECR, Tehran, Iran; 2 Department of Obstetrics and Gynaecology, Faculty of Medicine, Iran University of Medical Sciences, Tehran, Iran; 3 Department of Epidemiology and Reproductive Health, Reproductive Epidemiology Research Centre, Royan Institute for Reproductive Biomedicine, ACECR, Tehran, Iran

**Keywords:** Body Mass Index, Female, Intracytoplasmic Sperm Injections, Live Birth, Male

## Abstract

**Background:**

We designed the present study to evaluate the simultaneous effect of obesity in couples on in vitro fertilization/intracytoplasmic sperm injection (IVF/ICSI) outcomes.

**Materials and Methods:**

In this cross-sectional study, performed at Royan Institute between January 2013 and Janu- ary 2014, we evaluated the recorded data of all patients during this time period. The study population was limited to couples who underwent ICSI or IVF/ICSI cycles with autologous oocytes and fresh embryo transfers. We recorded the heights and weights of both genders and divided them into groups according to body mass index (BMI). Multilevel logistic regression analysis was used to determine the odds ratio for live births following ICSI or IVF/ICSI.

**Results:**

In total, 990 couples underwent IVF/ICSI cycles during the study period. Among the ovulatory women, a significant difference existed between the BMI groups. There was a 60% decrease [95% confidence interval (CI): 0.11-0.83] in the odds of a live birth among overweight subjects and 84% (95% CI: 0.02-0.99) decrease among obese subjects. Among the anovulatory women, the association between the BMI and live births presented no clear tenden- cies. We did not observe any significant relationship between male BMI and live birth rate. The results demonstrated no significant association between the couples’ BMI and live birth rate.

**Conclusion:**

Based on the present findings, increased female BMI independently and negatively influenced birth rates after ICSI. However, increased male BMI had no impact on live births after ICSI, either alone or combined with in- creased female BMI.

## Introduction

Obesity is an important risk factor for health problems and is deemed to be 1 of the 10 global diseases that contributes to an increased health burden. There is a rapidly increasing incidence of this complication in many industrialized countries, particularly the United States, and in developing Asian countries (1).

In numerous studies, researchers evaluated the effects of obesity on assisted reproductive technology (ART) cycle outcomes in women (2-13) and reported inconsistent results. Koning et al. (14) in a review article, reported that there were limited data despite 14 available studies in this area and concluded that further studies were needed to achieve an accurate insight.

Currently, there is no evidence to indicate that obesity increases the risk for ART complications; however, some researchers have reported the negative effects of obesity on pregnancy rates (14). In contrast, a review article published by Rittenberg et al.(15) reported an association between obesity and excess weight in women with poor pregnancy outcomes. This finding included reduced rates for clinical pregnancy and live births. Luke et al. (7) concluded that obesity had a negative impact on clinical pregnancy and live birth rates along with ART cycles with autologous oocytes. They emphasized that this risk could be brought under control by the use of donor oocytes.

The mechanism of the effects of female obesity on ART outcomes is controversial. The impact of obesity on ART outcomes in men is less studied (1, 16, 17) with conflicting results. A systemic review and meta-analysis by MacDonald et al. (18) published in 2010, has found no evidence of a relationship between increased body mass index (BMI) and semen parameters. Thus, further studies would be warranted in this field. Petersen et al. (19) reported that maternal and paternal BMI, both independently and combined, exerted negative effects on live birth rates after in vitro fertilization (IVF) cycles, but this association in intracytoplasmic sperm injection (ICSI) cycles was less obvious. In light of the current evidence, we designed the present study to assess the impacts of obesity in a couple on ART outcomes. This study sought to answer the question of whether obesity simultaneously in a couple has a negative effect on ICSI cycle outcomes in comparison to couples who have normal BMIs.

## Materials and Methods

This was a cross-sectional study performed at Royan Institute between January 2013 and January 2014. The Review Board and Ethics Committees of Royan Institute approved the study protocol. All participating couples provided ethical permission at their initial visit for the use of their treatment outcomes. Participant confidentiality for all participants was assured during the research and written informed consent was obtained from them.

### Subjects

**Fig.1:**
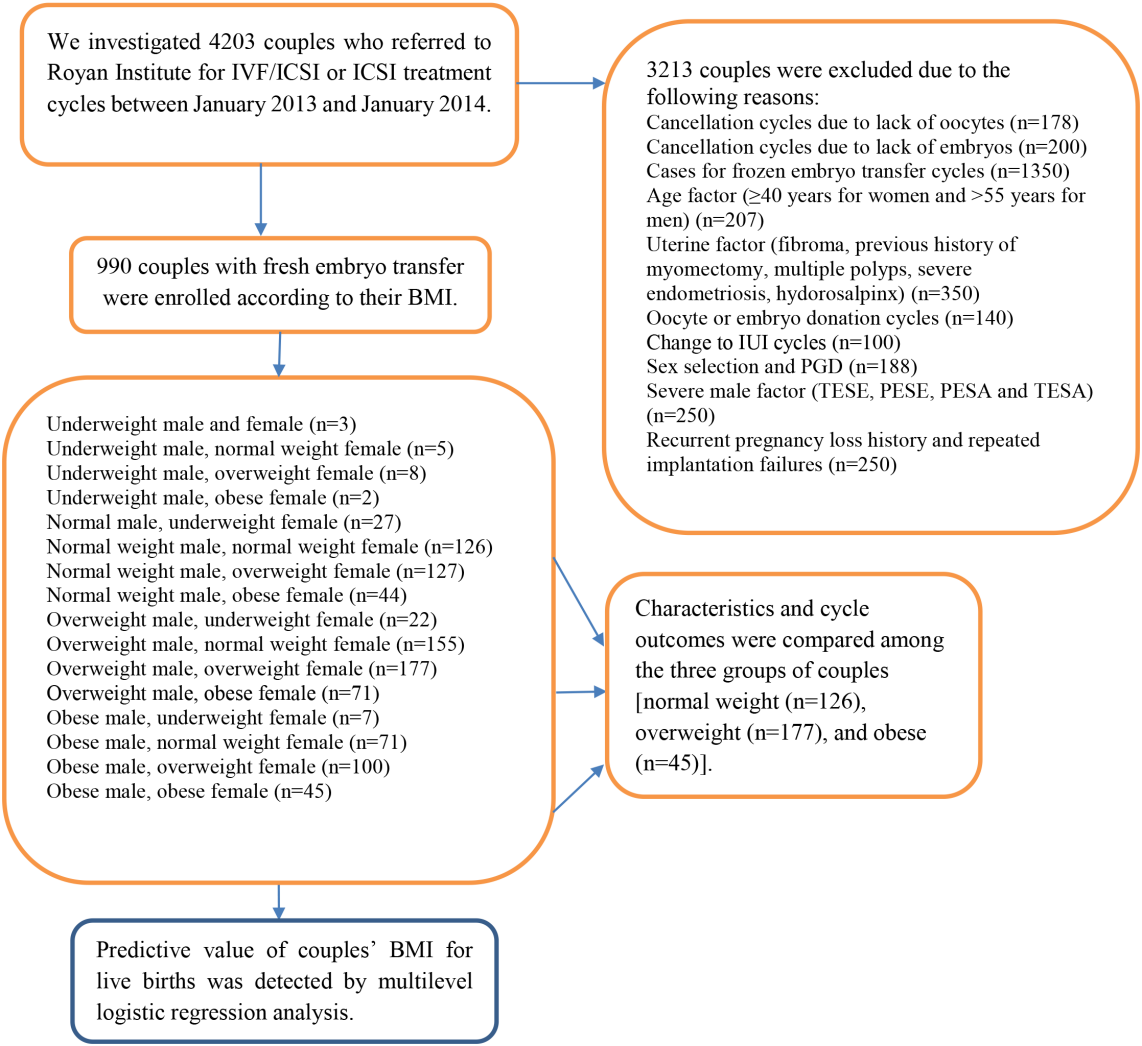
Sampling procedure and the distribution of the couples according to their BMI. IVF; In vitro fertilization, ICSI; Intra-cytoplasmic sperm injection, BMI; Body-mass index, IUI; Intrauterine insemination, PGD; Pre-gestational diagnosis, TESE; Testicular sperm extraction, PESE; Percutaneous epididymal sperm extraction, PESA; Percutaneous epididymal sperm aspiration, and TESA; Testicular sperm aspiration.

We evaluated the data recorded during the study period from all of the study participants. The study population was limited to patients who underwent ICSI or IVF/ICSI cycles that resulted in the transfer of 2 or 3 fresh embryos. Height and weight were recorded for all couples. Couples whose female partner was =39 years of age and the male partner was <55 years of age (17) at the time of the treatment cycle onset were enrolled to minimize the effect of age as a confounding factor. We excluded all cases with uterine factor, severe male factor, severe endometriosis, and gamete or embryo donor recipients (Fig .1). 

The patients’ age (years) was recorded at the beginning of treatment. At the onset of treatment, we classified participants as smokers or non-smokers according to the number of cigarettes smoked per day. The diagnosis of infertility was determined according to the 10th revision of the International Classification of Diseases (11). Accordingly, women participants were categorized as ovulatory or an ovulatory. Standard ovarian stimulation protocols were performed according to routine clinical practice. In brief, suppression of the endogenous luteinizing hormone surge was performed with either gonadotropin-releasing hormone agonists or antagonists. Controlled ovarian stimulation was performed with recombinant follicle-stimulating hormone (rFSH) and/or human menopausal gonadotropin (hMG); trans-vaginal ultrasound guided ovum pickup was performed 34-36 hours after administration of human chorionic gonadotropin (hCG). ICSI for retrieved MII oocytes, with or without insemination, was performed in accordance with standard general recommendations. 

We defined normal fertilization as the appearance of the 2^nd^ polar body at 16-19 hours after insemination or microinjection. In our institute, embryo quality is graded as A, B, C, and D, with "A" defined as the best quality and "D", the worst, according to cell numbers, percentage of fragmentation, and cell symmetry. All embryo transfers were performed with a Labotect catheter (Labotect, Germany) by experienced gynaecologists and embryologists on day 3 after IVF/ICSI. Luteal phase support was provided by administration of 400 mg of vaginal progesterone twice a day until the day of the ß-hCG test. Luteal support with progesterone was prescribed until the observation of foetal heart activity and subsequently tapered until week 8 of gestation. The main outcomes were fertilization, implantation, clinical pregnancy, and live birth rates. The implantation rate was denoted as the number of visualized intrauterine gestational sacs divided by the number of transferred embryos. A clinical pregnancy was documented by ultrasound observation of an intrauterine gestational sac with foetal cardiac activity. We defined spontaneous abortion as the loss of clinical pregnancy prior to 20 weeks gestation. Trained nurses routinely measured height and weight in participants of both genders prior to the onset of the treatment cycle. The balance scale for the measurement of weight was calibrated daily and verified by a one kg counterweight. We used the World Health Organization’s definition of BMI (kg/m^2^) to classify male and female participants as underweight (<18.5 kg/m^2^), normal (18.5-24.9 kg/m^2^), overweight (25-29.9 kg/m^2^), or obese (=30 kg/m^2^) (20). The small number of underweight couples precluded their inclusion in the couples’ analysis. We divided the couples into 3 groups based on male and female BMI results: group 1 (normal weight), group 2 (overweight), and group 3 (obese). The main outcomes were compared among the three groups. 

### Statistical analysis

Statistical analysis was carried out using the Statistical Package for the Social Sciences (SPSS), version 20.0 (SPSS Inc., Chicago, IL, USA). The study population’s characteristics were compared according to the couples’ BMI (normal, overweight, and obese) using one-way analysis of variance (ANOVA), Kruskal- Wallis nonparametric analysis of variance, and the chi-square test when appropriate. Multilevel logistic regression analysis was applied to determine the odds of live births following ICSI cycles. The analysis was conducted according to the female and male BMI groups. Normal-weight patients were considered to be the reference group. Analysis of female BMI was adjusted for age and duration of infertility. Likewise, analysis of the male BMI was adjusted for age, duration of infertility, and smoking status. 

A multilevel logistic regression analysis was used to detect the predictive factors for live births after ICSI cycles. All possible factors that affected the live birth rate, which included female and male ages, couples’ BMI (<25 kg/m^2^ and =25 kg/m^2^), male smoking status, cause and duration of infertility, ovarian stimulation protocol [long gonadotropin-releasing hormone (GnRH) agonist and GnRH antagonist protocols], and number and quality of transferred embryos were incorporated into the model. The results of the multilevel logistic regression analysis have been presented as adjusted odds ratios (ORs) with 95% confidence intervals (CIs). P values <0.05 were considered statistically significant.

## Results

In total, there were 4203 ART cycles during the study period. A total of 990 eligible women and their husbands underwent 927 ICSI and 63 ICSI with insemination (IVF/ICSI) cycles according to the inclusion criteria. The sampling procedure and distribution of the couples according to their BMI has been illustrated in (Fig .1). According to BMI, of the 990 women participants, there were 59 (6%) underweight, 357 (36%) with normal weight, 412 (41.6%) overweight, and 162 (16.4%) obese participants. Of the 990 men evaluated, there were 18 (1.8%) underweight, 325 (32.7%) normal weight, 425 (43%) overweight, and 223 (22.5%) obese participants.

The characteristics of the study population according to gender and BMI have been presented in (Table 1). The distribution of smoking in the males significantly differed among the BMI groups (P=0.006). The majority of females (n=786, 79.3%) had normal menses and ovulation. As expected, there were more anovulatory cases in the obese group than in the other groups (P=0.003).

Anovulatory cases in the present study consisted of participants with PCOS (n=153, 75%) and age factor (over 37 to 39 years, n=51, 25%). 

**Table 1:** Basic characteristics of the studied population according to gender and body mass index


Characteristics		Underweight	Normal weight	Overweight	Obese	P value

		n=59	n=357	n=412	n=162	
Women						
	Age (Y)	29.0 ± 3.9	30.0 ± 4.5	30.7 ± 4.8	32.2 ± 4.9	<0.001
	Anovulatory cases	11 (18.6)	59 (16.5)	84 (20.4)	50 (30.9)	0.003
		n=45	n=297	n=425	n=223	
Men						
	Age (Y)	35.1 ± 6.7	34.8 ± 5.6	35.0 ± 5.7	36.0 ± 6.4	0.081
	Smoking cases	21 (35.5)	58 (17.7)	88 (21.3)	69 (42.5)	0.006


Data are presented as mean ± SD or n (%).

We separately evaluated the impact of female and male BMI on the live birth rate in ICSI. The results of the multilevel regression analysis according to female and male BMI has been shown in (Table 2). Among the ovulatory women, there was a significant difference between the BMI groups, with a 60% (95% CI: 0.11-0.83) decrease in the odds for live birth among overweight individuals and 84% (95% CI: 0.02-0.99) decrease in the odds for live birth among obese individuals. Trend analysis showed a significant reduction of 9% (95% CI: 0.83-0.99) with each one unit increase in BMI (P=0.04). Among anovulatory women, the association between BMI and live births presented no clear tendencies, even though the ORs indicated lower probabilities for live births among overweight and obese anovulatory women. The 95% CIs were not significant. Among anovulatory women, the trend analysis showed a significant reduction of 15% (95% CI: 0.72- 0.98) with every one unit increase in BMI (P=0.02). In both ovulatory and anovulatory underweight women, we observed a significant elevation in the odds of live births of 6.5 times (95% CI: 2.1-20.65) and 7.3 times (95% CI: 0.99-55.1), but the CIs were too wide because of the low sample size. The results for men participants presented no significant relationship between BMI and live births. 

The comparison of the three groups of couples according to BMI has been demonstrated in (Table 3). The overweight (P=0.01) and obese (P<0.001) couples were significantly older than normal weight couples. The results indicated that the three groups were comparable in terms of type, cause of infertility, number of previous ART cycles, and stimulation outcomes. There were no significant differences between the three groups in terms of fertilization, implantation, clinical pregnancy, multiple pregnancy, miscarriage, and live birth rates. 

**Table 2:** Multilevel logistic regression analyses of the probability for live births following all ICSI or IVF/ICSI cycles according to gender and stratified by BMI


Variables		n (%)	OR^a^	95% CI	P value^b^

Female^b^ BMI, (n=990 ICSI cycles among 990 women)					
Ovulatory					
	Underweight	48 (6.1)	6.5	(2.1-20.65)	0.001
	Normal weight	298 (37.9)	Reference group	-	-
	Overweight	328 (41.0)	0.30	(0.11-0.83)	0.021
	Obese	112 (14.2)	0.14	(0.02-0.99)	0.049
	Trend	786 (100)	0.91	(0.83-0.99)	0.045
Anovulatory					
	Underweight	11 (5.3)	7.30	(0.99-55.1)	0.050
	Normal weight	59 (28.9)	Reference group	-	-
	Overweight	84 (41.1)	0.50	(0.10-2.4)	0.392
	Obese	50 (24.5)	0.14	(0.009-2.4)	0.186
	Trend	204 (100)	0.85	(0.72-0.98)	0.021
Male^b^ BMI, (n=990 ICSI cycles among 990 men)					
	Underweight	45 (4.5)	0.295	(0.02-3.96)	0.352
	Normal weight	297 (30.0)	Reference group	-	-
	Overweight	425 (42.9)	0.07	(0.0-10.71)	0.360
	Obese	223 (22.5)	0.012	(0.0-21.89)	0.294
	Trend	990 (100)	1.01	(0.95-1.09)	0.647


OR; Odds ratio, CI: Confidence interval, BMI; body mass index, IVF; In vitro fertilization, ICSI; Intra-cytoplasmic sperm injection, ^a^; ORs with 95% CIs and P values from Wald tests, and ^b^; Female analyses adjusted for age and duration of infertility. Male analyses adjusted for age, duration of infertility, and smoking status.

**Table 3:** Comparison of study population characteristics and cycle outcomes among three groups of couples according to BMI


Variable		Normal weight couplesn=126	Overweight couplesn=177	Obese couples n=45	Test	P

Female age (Y)		30.0 ± 4.7	30.5 ± 4.6	32.3 ± 4.7	ANOVA	0.018ᵃ
Male age (Y)		33.4 ± 4.7	35.1 ± 5.9	37.9 ± 5.4	ANOVA	<0.001ᵇ
Female FSH (IU/l)		6.8 ± 3.6	6.1 ± 3.0	6.9 ± 3.4	ANOVA	0.160
Female LH (IU/l)		4.7 ± 3.3	4.6 ± 4.1	4.6 ± 4.7	ANOVA	0.986
Female AMH (ng/ml)		2.3 ± 1.7	2.4 ± 1.6	2.0 ± 1.4	Kruskal-Wallis	0.646
Female TSH (mIU/l)		2.1 ± 1.8	2.3 ± 1.9	2.3 ± 1.7	ANOVA	0.658
Type of infertility					Chi-square	0.426
	Primary	107 (84.9)	157 (88.7)	37 (82.3)		
	Secondary	19 (15.1)	20 (11.3)	8 (17.7)		
Cause of infertility					Chi-square	0.150
	Ovulatory	105 (83.3)	32 (71.1)	147 (83.1)		
	Anovulatory	21 (16.7)	13 (28.9)	30 (16.9)		
Male factor infertility cases		89 (70.6)	30 (66.7)	131 (74)	Chi-square	0.577
Infertility duration (Y)		5.1 ± 3.4	6.39 ± 4.7	7.08 ± 4.7	ANOVA	0.011^c^
Number of previous ART cycles		0.4 ± 0.9	0.3 ± 0.6	0.4 ± 0.9	ANOVA	0.583
Stimulation duration (days)		10.7 ± 2.0	10.6 ± 2.1	10.9 ± 2.4	ANOVA	0.664
Total amount of rFSH dose (IU)		1932.6 ± 724.0	1810.1 ± 821.8	1839.9 ± 844.4	Kruskal-Wallis	0.176
Total dose of used gonadotropins (IU)		2086.1 ± 1005.4	2087.9 ± 903.8	2251.6 ± 1206.3	ANOVA	0.582
Stimulation protocol					Chi-square	0.564
	Long agonist	108 (85.7)	40 (88.9)	149 (84.2)		
	Antagonist	18 (14.3)	5 (11.1)	28 (15.8)		
Total number of retrieved oocytes		8.9 ± 4.3	8.3 ± 3.4	8.0 ± 3.52	ANOVA	0.327
Total number of embryos		5.1 ± 2.7	5.0 ± 2.7	5.0 ± 2.50	ANOVA	0.974
Number of transferred embryo		2.4 ± 0.56	2.4 ± 0.5	2.4 ± 0.55	ANOVA	0.978
Endometrial thickness on transfer day (mm)		9.9 ± 1.4	9.9 ± 1.6	9.5 ± 1.9	ANOVA	0.293
Quality of transferred embryos (ET)^*^					Chi-square	0.675
	Good	82 (65.1)	111 (62.7)	32 (71.1)		
	Fair	11 (8.7)	20 (11.3)	2 (4.4)		
	Poor	33 (26.2)	46 (26)	11 (24.4)		
Fertilization rate		0.74 ± 0.23	0.73 ± 0.24	0.78 ± 0.21	ANOVA	0.523
Implantation rate		0.28 ± 0.173	0.31 ± 0.2	0.33 ± 0.16	ANOVA	0.593
Clinical pregnancy rate		40 (78.4)	68 (89.5)	21 (87.5)	Chi-square	0.407
Blighted ovum		9 (17.6)	6 (7.9)	2 (8.3)	Chi-square	0.231
Ectopic pregnancy rate		2 (3.9)	2 (2.6)	1 (4.2)	Chi-square	0.936
Multiple pregnancy rate		7 (19.4)	15 (27.3)	5 (27.8)	Chi-square	0.664
Miscarriage rate		2 (5.3)	9 (14.1)	2 (10)	Chi-square	0.362
Live birth rate		36 (94.7)	55 (85.9)	18 (90)	Chi-square	0.345


Data are presented as mean ± SD or n (%). BMI; Body mass index, FSH; Follicle stimulating hormone, LH; Luteinizing hormone, AMH; Anti-Müllerian hormone, TSH; Thyroid stimulating hormone, ART; Assisted reproductive technology, rFSH; Recombinant follicle-stimulating hormone, ANOVA: One-way analysis of variance, ^a^; Obese couples vs. overweight couples (P=0.015), normal BMI vs. obese couples (P=0.040) according to Tukey’s test, ^b^; Normal BMI vs. overweight couples (P=0.013), normal BMI vs. obese couples (P<0.001), overweight vs. obese couples (P=0.086) according to Tukey’s test, ^c^; Normal BMI vs. overweight couples (P=0.032), normal BMI vs. obese couples (P=0.021) according to Tukey’s test, *; Good quality embryos-all ET were A, B, or AB, Fair-half of ET were good quality (AC, BC), Poor quality-all ET were C, D, or CD.

The results of the multilevel logistic regression model for the detection of the predictive factors for the live birth rate showed that none of the included variables remained in the final model as significant factors. The results also revealed no significant association between the couples’ BMI and live births (Table 4).

**Table 4:** Multilevel logistic regression analysis for detection of predictive factors for live birth after ICSI or IVF/ICSI cycles in the studied population


Combined BMI (kg/m^2^)			Live birth per ICSI cycle		
Women	Men	n (%)	OR	95% CI	P value

<25	<25	161 (16.3)	1	Reference group	-
<25	≥25	255 (25.8)	1.03	0.5-1.9	0.914
≥25	<25	181 (18.3)	0.6	0.3-1.2	0.262
≥25	≥25	393 (39.7)	0.8	0.4-1.9	0.864


ICSI; Intracytoplasmic sperm injection, IVF; In vitro fertilization, BMI; Body mass index, OR; Odds ratio, and CI; Confidence interval.

## Discussion

Previous studies separately evaluated the effects of both genders’ BMI on ART outcomes. The synergistic effects of obesity in couples were reported in limited studies (8, 19). We have excluded the main confounding factors that affect live birth rates in order to accurately assess the independent effects of a couple’s obesity on ART outcomes. Our results revealed that a couple’s BMI had no effect on the outcomes of ICSI with fresh cleavage-stage embryo transfer cycles.

Our results supported those published in 2013 by Petersen et al. (19), who reported that the combined increased maternal and paternal BMI had no significant effect on live birth rate in ICSI cycles. However, the authors have presented the negative impacts of increased female and male BMI, both individually and combined, on live births in IVF cycles. In our institute, treatment cycles with only IVF are uncommon and the majority of treatment cycles include ICSI or IVF/ICSI procedures. Therefore, we could not evaluate these subjects according to IVF cycles. 

The effects of female BMI on ART outcomes were evaluated in several studies. Our findings showed that among ovulatory women, BMI had a negative impact on live births. In anovulatory women, we observed a tendency for less odds of live births in the obese group, which was not statistically significant. Therefore, our results agreed with some recent studies where female BMI negatively impacted ART outcomes (8, 11, 13, 15, 20). On the other hand, previous studies indicated no negative effect of female BMI on ART outcomes (2, 12, 21-23). Petersen et al. (19) demonstrated that the female BMI had a negative impact on live birth rates in IVF cycles, but this was less clear in ICSI cycles. A prospective study conducted by Chavarro et al. (24) evaluated 170 women who underwent 233 ART cycles and found an association between overweight and obese women with decreased live birth rates. Moragianni et al. (25), in a retrospective research of 4609 patients, found that obesity had significant negative effects on ART outcomes, with up to 68% lower odds of live births following the first ART cycle. Rittenberg et al. (15), in a meta-analysis of 47967 IVF/ICSI cycles, reported that an increased female BMI was aligned with adverse pregnancy outcomes in IVF/ICSI treatment cycles and this effect was observed in both overweight and obese women. Since the earlier investigations did not categorize their findings according to type of treatment cycle (IVF or ICSI), a more adverse influence of increased BMI in IVF compared to ICSI might have been concealed and the intensity of the BMI impact on IVF/ICSI possibly relied on the IVF and ICSI cycle distributions in the sample size (19). Although a number of multiparous women are obese, a negative association of obesity with women’s reproductive health has been reported (26). Because of the conflicting results reported by studies, the mechanism action of maternal obesity on IVF or IVF/ICSI outcomes remains unclear (27). Although a number of oocyte donation studies have suggested negative effects of obesity on the endometrium (6, 28), others have not (2, 21, 29). Endocrine changes related to obesity such as hyperandrogenism and insulin resistance as well as alterations in the local insulin-like growth factors (IGFs), cytokines, and leptin levels may play a major role in the adverse effects of an increased BMI on ART outcomes (4). According to previous studies (13), the mechanism of action of obesity in anovulatory cases, especially PCOS women, is different and depends on the intensity of the endocrine changes.

The influence of male BMI on ART outcomes has been less studied. The existing literature contains only 7 studies on this subject (1, 8, 11, 16, 17, 19, 30). The first study, published in 2011 by Bakos et al. (1), reported an association between high paternal BMI with significantly reduced clinical pregnancy and live birth rates after ART. Two recent studies presented that male BMI was associated with a negative impact on clinical pregnancy and live birth rates after IVF, but not after ICSI. Additionally, the previous studies in this field reported that ICSI might overcome the negative impact of obesity on sperm-oocyte interaction (16, 19). On the other hand, a prospective study conducted by Colaci et al. (17) evaluated 114 couples who underwent 172 ICSI cycles and concluded that male obesity was associated with decreased odds for live births after ICSI. Our results indicated that the male BMI had no effect on live birth rates after ICSI. The deleterious effects of male obesity could be due to an altered hormonal profile and decreased semen quality related to increased leptin and E2 levels, and disturbance in spermatogenesis (19, 31). However, a systematic review with meta-analysis found no evidence of an association between an increased BMI and semen parameters (32). A systematic review by Campbell et al. (33) in 2015 reported that the rate of births per ART cycle was reduced by 35% in obese men. The salient weak point in the previous studies and our study was the use of BMI as a marker of body fat in men. In view of these conflicting results, we suggest that prospective studies should evaluate the effects of male and female abdominal obesity on reproductive parameters and ART outcomes via other anthropometric measurements (waist and hip circumferences). Currently, the role of the male BMI in ART processes and outcomes is partly understood. Further investigations are needed to arrive at reliable conclusions (13).

We analysed the couples and found no synergistic negative impact of increased female and male BMI on live births after ICSI cycles. This finding agreed with studies by Petersen et al. (19) and Schliep et al. (34). Some studies assessed the effects of combined male and female BMI on ART outcomes (4, 8, 10, 19). Petersen et al. (19) evaluated the effects of parental BMI on live birth rates after ART cycles. They reported that increased combined female and male BMI had a negative impact on live birth rates after IVF cycles; however, its effects in terms of ICSI were less clear. Schliep et al. performed a prospective assessment of 721 couples and found no influence of the couples’ weight status on IVF outcomes (34). In contrast, a recent study by Wang and colleagues retrospectively investigated 12061 first fresh IVF/ICSI cycles and reported that female obesity exerted negative effects on live births after IVF; nonetheless, there was no evidence of a negative impact by the parental BMI on ICSI outcomes (4). In contrast, Umul et al. (10) found that couples’ obesity had a negative impact on clinical pregnancy rates and live birth rates following ICSI cycles. In the present study we meticulously analysed the characteristics of the couples in ICSI cycles and adjusted the impact of confounding factors on our results. Recent data have confirmed the findings of those previous studies that reported no significant influence of the parental BMI on ICSI success. In view of the conflicting results, we suggest that more research be undertaken to shed sufficient light on this issue.

The present study has some limitations. There was no data about the specific hormonal profile and android or gynoid distribution of fat in anovulatory and ovulatory women, and no data about semen analysis parameters to compare among different BMI groups. We propose that these parameters should be considered in future studies.

## Conclusion

Based on the current findings, an increased maternal BMI independently influenced negatively live birth rate after ICSI cycles, whereas increased paternal BMI separately and in combination with maternal BMI did not show this affect.
